# Lung Cancer Mortality and In-Hospital Outcomes in Chile: A Population-Based Cross-Sectional Study

**DOI:** 10.3390/healthcare14213688

**Published:** 2026-10-31

**Authors:** Iris Delgado, Yanara A. Bernal, Carla Campaña, Macarena R. Vial, Claudia Asenjo-Lobos, Cecilia González, Andrea Olea, Gabriela Meza-Fuentes, Rene López, Alex Godoy-Faúndez, Diego Rivera, Diego Carrillo

**Affiliations:** 1 Centro de Epidemiología y Políticas de Salud, Facultad de Medicina Clínica Alemana, Universidad del Desarrollo, Santiago 7610658, Chile; idelgado@udd.cl (I.D.); aolea@udd.cl (A.O.); 2 Instituto de Ciencias e Innovación en Medicina (ICIM), Facultad de Medicina Clínica Alemana, Universidad del Desarrollo, Santiago 7610658, Chile; cgonzalezcaro@udd.cl (C.G.); gmezaf@udd.cl (G.M.-F.); 3 School of Nursing, Pontificia Universidad Católica de Chile, Santiago 7820436, Chile; 4 Centro para la Prevención y el Control del Cáncer (CECAN), Facultad de Medicina, Universidad de Chile, Santiago 8380453, Chile; ccampana@udd.cl; 5 Center of Global Intercultural Health (CeSGI), Facultad de Medicina Clínica, Alemana Universidad del Desarrollo, Facultad de Psicología, Universidad del Desarrollo, Santiago 7610658, Chile; 6 Interventional Pulmonology Unit, Clinica Alemana de Santiago, Universidad del Desarrollo, Santiago 7610658, Chile; mrodriguezvi@alemana.cl; 7 Centro de Estudios Clínicos, Instituto de Ciencias e Innovación en Medicina (ICIM), Facultad de Medicina Clínica Alemana, Universidad del Desarrollo, Santiago 7610658, Chile; casenjo@udd.cl; 8 Grupo Intensivo, Instituto de Ciencias e Innovación en Medicina (ICIM), Facultad de Medicina Clínica Alemana, Universidad del Desarrollo, Santiago 7610658, Chile; rene.lopez@udd.cl; 9 Departamento de Paciente Crítico, Clínica Alemana de Santiago, Santiago 7610658, Chile; 10 Centro de Investigación en Sustentabilidad (CiSGER), Facultad de Ingeniería, Universidad del Desarrollo, Santiago 7610658, Chile; alexgodoy@udd.cl (A.G.-F.); diegorivera@udd.cl (D.R.); 11 Departamento de Hemato-Oncología, Facultad de Medicina, Pontificia Universidad Católica de Chile, Santiago 8331150, Chile; dicarrillo@uc.cl

**Keywords:** administrative health data, in-hospital mortality, age-standarized mortality, hospital discharge, ICD-10, sex differences, pneumonia

## Abstract

**Highlights:**

**What are the main findings?**

- Lung cancer mortality in Chile showed diverging trends by sex: age-adjusted mortality decreased by 35.5% among men but increased by approximately 9% among women between 1997 and 2024.
- Pneumonia and acute respiratory complications were associated with approximately twofold higher odds of in-hospital death among patients hospitalized with lung cancer.

**What are the implications of the main findings?**

- The observed association between pneumonia and in-hospital mortality highlights the clinical relevance of respiratory infection prevention and management among patients with lung cancer.
- Early recognition and timely management of respiratory complications may be clinically relevant in hospitalized patients with lung cancer.

**Abstract:**

Background/objective: Lung cancer (LC) remains the leading cause of cancer-related death worldwide. However, population-level evidence on long-term mortality trends and hospital outcomes in Latin America remains limited. This study aimed to analyze long-term mortality trends from LC in Chile and characterize hospitalization patterns and factors associated with in-hospital mortality. Methods: We conducted a population-based cross-sectional study using two independent administrative databases. Population-level lung cancer mortality trends were analyzed using national mortality records from DEIS (1997–2024), whereas hospitalization patterns and in-hospital mortality were analyzed using hospitalization episodes from the GRD-FONASA database (2019–2024). LC deaths were identified using ICD-10 codes C34. Hospitalizations were analyzed by demographics, primary diagnosis, and discharge status. Logistic regression models were used to evaluate factors associated with in-hospital mortality. Results: Age-adjusted mortality declined substantially among men (−35.5%) but increased slightly among women (+9%). Among hospitalization episodes recorded in the GRD-FONASA database, in-hospital mortality was 17.4%. In-hospital mortality was higher among men than women (18.7% vs. 15.8%), and male sex remained associated with increased odds of in-hospital death after adjustment for age (aOR 1.22; 95% CI 1.13–1.31). Lung cancer or metastasis was the principal diagnosis in 10,986/14,778 hospitalizations (74.3%) included in the selected diagnostic analysis. However, pneumonia and acute respiratory complications were disproportionately more frequent among hospitalizations ending in death. In multivariable analysis, pneumonia (aOR 2.05; 95% CI 1.77–2.38) and acute respiratory complications (aOR 2.13; 95% CI 1.83–2.48) were independently associated with in-hospital death. Conclusions: LC mortality trends in Chile diverged by sex, with declining age-adjusted mortality among men but increasing mortality among women. Respiratory infections and acute respiratory complications were strongly associated with in-hospital mortality, highlighting their importance as key targets for prevention and clinical management.

## 1. Introduction

Lung cancer is the leading cause of cancer-related deaths worldwide, accounting for more than 1.8 million deaths annually [1]. However, despite advances in prevention, diagnosis and treatment, the prognosis remains poor, mainly because most cases are diagnosed at advanced stages of the disease [2]. Trends in lung cancer mortality have shown significant changes over recent decades, particularly in relation to sex. In many high-income countries, a sustained decline in mortality among men has been observed, a phenomenon attributed mainly to the reduction in smoking rates that began several decades ago [3]. In contrast, trends among women have been more variable, with increases or stabilization in mortality in several countries, likely reflecting the historical delay in the onset of tobacco use and in the implementation of tobacco control policies targeting this population [4]. These temporal differences in exposure patterns have led to a progressive shift in the burden of lung cancer mortality between men and women [5].

In Latin America, evidence on lung cancer mortality trends remains limited compared with other regions of the world [6]. Chile has one of the highest cancer mortality rates in the region, and lung cancer is a major cause of death in both men and women [1]. However, studies assessing long-term mortality trends and sex differences in the Chilean context remain scarce. Understanding these trends is essential for guiding lung cancer prevention and control strategies, including tobacco control policies [7].

Beyond mortality, lung cancer is associated with a high burden of hospital morbidity, particularly due to acute pulmonary complications that account for a substantial share of admissions and in-hospital deaths [6,8,9,10]. Acute pulmonary complications represent an important cause of hospitalization in this population; however, their burden in population-based studies, particularly in middle-income countries, remains poorly characterized. The combined use of national mortality records and the FONASA hospital discharge database (GRD) enables a population-based analysis of lung cancer from two complementary perspectives: long-term mortality trends and hospitalization patterns among patients with this disease [11].

Therefore, the objective of this study was to analyze trends in lung cancer mortality in Chile over the past few decades and to describe recent hospitalization patterns among patients with this disease, with a particular focus on respiratory infections and other acute pulmonary complications. In addition, differences by sex and age were evaluated, as well as factors associated with in-hospital mortality. This analysis provides relevant population-based evidence for understanding the evolution of lung cancer in Chile and for identifying clinical complications associated with higher odds of death during hospitalization.

## 2. Materials and Methods

### 2.1. Study Design

A population-based retrospective study was conducted using two independent administrative data sources from Chile: the national mortality registry (DEIS, 1997–2024) and the FONASA hospital discharge database (GRD, 2019–2024). The two databases were analyzed separately and were not linked at the individual level. DEIS data were used to assess national population-level lung cancer mortality trends, whereas GRD-FONASA data were used to characterize hospitalization episodes and in-hospital outcomes among FONASA beneficiaries with lung cancer. Therefore, the mortality and hospitalization analyses represent distinct study populations.

### 2.2. Data Sources

Mortality database: Mortality data were obtained from the National Vital Statistics System, which includes all deaths in our country from 1997 to 2024. This database compiles information from medical death certificates registered nationwide (Department of Health Statistics and Information, DEIS). It contains demographic and clinical data, including year of death, sex, age at death, and underlying cause of death coded according to the International Classification of Diseases, 10th Revision (ICD-10). Deaths attributed to lung cancer were identified using ICD-10 codes corresponding to malignant neoplasms of the bronchi and lung (C34). Due to its national coverage and the standardization of cause-of-death coding, this registry constitutes a robust source for epidemiological analysis of mortality. Age-standardized mortality rates were calculated using direct standardization, with the 2024 Chilean population used as the standard population. Temporal trends in age-adjusted mortality rates were assessed using log-linear regression for the period 2002–2024. Annual percentage change (APC) and corresponding 95% confidence intervals (CIs) were estimated for the overall population and separately by sex. Mortality was additionally projected through 2035 using the Nordpred age-period-cohort framework [12]. Projections were generated separately for men and women using observed mortality and population data and are reported as numbers of deaths and crude mortality rates for 2026–2030 and 2031–2035. The projected trend was left unattenuated for 2026–2030 and reduced by 25% for 2031–2035 to limit the influence of long-term extrapolation.

Hospital Discharge Database (Diagnosis-Related Groups [DRGs]): Hospitalization data were obtained from the Chilean National Diagnosis-Related Groups (DRG) database maintained by the National Health Fund (FONASA) from https://datosabiertos.fonasa.cl/ (accessed on 31 May 2025). This administrative database compiles clinical and hospital discharge information from hospitals using the DRG system across the public healthcare network, covering virtually all hospitalizations financed by the Chilean public health insurance system. This database contains data from 2019 to 2024 and includes the following variables: year of hospitalization, sex, age, discharge status, primary diagnosis (diag1), and up to 35 secondary diagnoses (diag2–diag35), all coded according to the ICD-10 classification. Hospitalizations related to lung cancer were identified by the presence of ICD-10 code C34 (malignant neoplasms of the bronchi or lung) in any of the diagnostic fields (diag1–diag35). For each hospitalization, information was extracted on demographic characteristics, diagnostic profile, and hospital discharge outcome. This database made it possible to characterize the clinical burden associated with hospitalizations of patients with lung cancer, with particular emphasis on the presence of respiratory infections and in-hospital mortality. Infectious diagnoses were identified across all diagnostic fields and grouped into clinically relevant categories for analysis. The database includes an encrypted patient identifier (CIP_ENCRIPTADO), which allowed for the identification of multiple hospitalizations for the same individual. However, the primary unit of analysis in this study was the hospitalization episode rather than the individual patient.

### 2.3. Study Population

For the mortality analysis, the study population included all deaths recorded in Chile where the underlying cause of death was lung cancer (ICD-10 C34) during the study period (1997–2024). Meanwhile, for the hospitalization analysis, the study population included all hospital discharges with a diagnosis of lung cancer during the study period. The unit of analysis was the hospitalization episode; therefore, a single patient could contribute more than one record if they had multiple hospitalizations during the study period. The primary outcome for hospitalization analysis was in-hospital mortality, defined as a discharge status recorded as death. The main exposure variables included sex, age, and the primary diagnostic category associated with hospitalization.

### 2.4. Variables

For mortality, the following variables were analyzed: year of death, sex, age at death, and cause of death. For descriptive analyses, age was also categorized into age groups. The hospital discharge database included the variables year of discharge, sex, age, discharge status (death or discharge), primary diagnosis, and secondary diagnoses. Diagnoses indicative of metastatic disease were identified using ICD-10 codes corresponding to secondary malignant neoplasms. Additionally, the main reason for hospitalization was classified into clinically relevant categories based on the recorded primary diagnosis (diag1). These categories were defined by considering both the clinical relevance and the frequency of the most common diagnoses observed in the database (top 5 primary diagnoses). For the principal-diagnosis analysis, hospitalization episodes were restricted to those whose primary diagnosis belonged to one of the prespecified diagnostic categories described above. Of the 19,186 lung cancer-related hospitalization episodes identified during the study period, 14,778 met this criterion and were included in the analysis. Hospitalizations with other principal diagnoses were excluded from this diagnostic-category comparison. Diagnoses corresponding to lung cancer and metastatic disease were grouped into the “Tumor/Metastasis” category (reference category), including ICD-10 codes such as C34, C78, and C79. Hospitalizations associated with pneumonia were grouped under the “Pneumonia” category, corresponding to codes J15 and J18. Acute respiratory complications were grouped into the “Complications” category, including codes for respiratory failure (J96), pleural effusion (J90), and pulmonary embolism (I26). The remaining diagnoses were grouped into additional categories such as “Other chronic obstructive pulmonary diseases” (J44) and “Other medical care” (Z51), to ensure analytical consistency and allow for comparison between clinically relevant groups. The complete ICD-10 mapping used to define the diagnostic categories is provided in Supplementary Table S1.

### 2.5. Missing Data

Diagnostic variables were coded in a binary manner, assigning a value of 1 to the presence of the diagnosis and 0 to its absence. The absence of a corresponding diagnostic code was interpreted as the condition not being recorded for that hospitalization episode and was therefore not considered missing data. For demographic variables, records with sex classified as “unknown” were excluded from the analyses. Age was calculated based on the difference between the date of hospital admission and the recorded date of birth.

### 2.6. Statistical Analysis

Age- and sex-adjusted mortality rates were calculated, and descriptive analyses were performed to characterize mortality patterns and the characteristics of hospitalizations among patients with lung cancer. Categorical variables were summarized using absolute frequencies and percentages, while continuous variables were described using means and standard deviations. In the hospitalization database, comparisons were made according to sex and discharge status. Differences in categorical variables were assessed using chi-square tests, while differences in continuous variables were analyzed using Student’s *t*-tests for independent samples when appropriate. To assess the association between sex and in-hospital mortality in patients hospitalized with lung cancer, a logistic regression model was fitted that included sex and age as independent variables. The tumor/metastasis category was selected as the reference because it represented hospitalizations primarily attributed to the underlying malignant disease, providing a clinically relevant comparator for hospitalizations associated with respiratory and other diagnostic categories. The original regression models used profile-likelihood 95% confidence intervals and model-based Wald *p* values. The supplementary sensitivity analyses used HC1 robust standard errors clustered by encrypted patient identifier to account for repeated hospitalization episodes. Given the clinical heterogeneity of the acute respiratory complications category, a sensitivity analysis replaced this combined category with separate principal-diagnosis categories for respiratory failure (J96), pleural effusion (J90), and pulmonary embolism (I26). A single logistic regression model was adjusted for sex, age, and calendar year, with patient-clustered robust standard errors. An additional sensitivity analysis excluded hospitalizations occurring in 2020–2021. Models adjusted for sex, age, principal-diagnosis category, and calendar year were compared with the corresponding full-period model; these supplementary models did not include readmission. Readmission was evaluated using logistic regression adjusted for age and sex. Additional models included principal-diagnosis category and were fitted overall and separately for women and men. Sex-specific models included age, readmission status, and principal-diagnosis category. First hospitalization and lung cancer or metastasis were the reference categories for hospitalization sequence and diagnostic group, respectively. Results were reported as odds ratios (OR) with 95% confidence intervals. No automated variable-selection procedure was used. Age was included as a continuous variable, assuming a linear association with the log odds of death, with ORs expressed per one-year increase. During revision, model convergence, model-matrix rank, and outcome counts within diagnostic categories were reviewed. The female Z51 estimate was reported as not estimable because no deaths occurred in this category, resulting in separation. The male Z51 estimate, based on one death, was flagged as unstable and was not interpreted as evidence of a protective effect. Analyses were performed using R version 4.4.3. All tests were two-sided, with *p* < 0.05 considered statistically significant.

### 2.7. Ethical Considerations

This study was conducted in accordance with the ethical principles outlined in the Declaration of Helsinki and its subsequent amendments. The analyses were based exclusively on anonymized secondary databases derived from national administrative records, including mortality and hospital discharge registries. These datasets do not contain direct personal identifiers and were accessed in an aggregated and de-identified format. Because the study involved the secondary analysis of routinely collected administrative data, without direct contact or interaction with participants and without the possibility of individual identification, it was considered a minimal-risk research project under current regulations. Consequently, informed consent was not required. All data were handled in accordance with applicable regulations regarding privacy, confidentiality, and the protection of personal information.

## 3. Results

### 3.1. Lung Cancer Mortality

**Figure 1:**
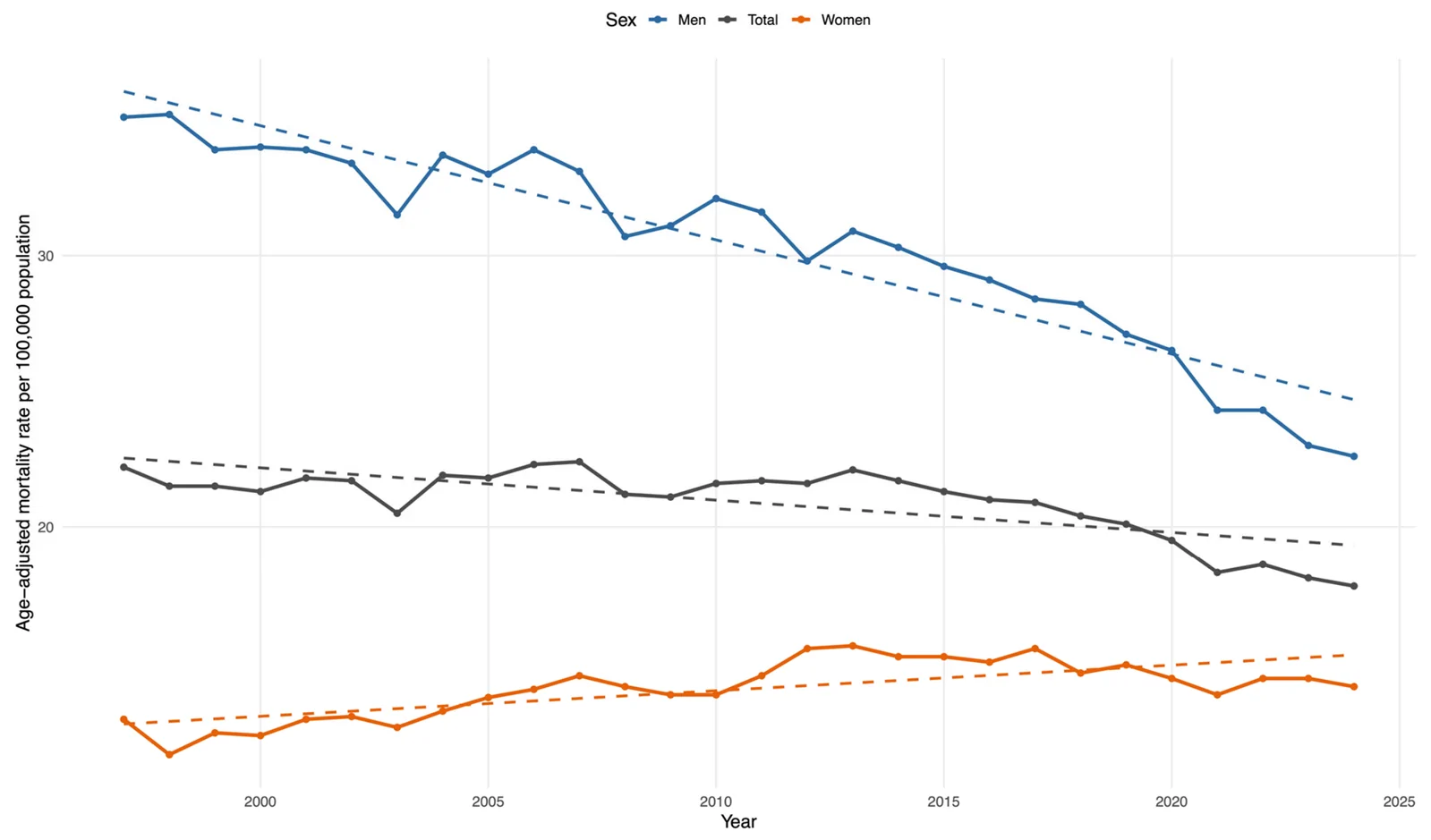
Age-adjusted lung cancer mortality rates in Chile by sex, 1997–2024. Age-adjusted mortality rates (per 100,000 population) for lung cancer (ICD-10 C34) in Chile between 1997 and 2024, stratified by sex. Rates were standardized using the national population structure. While age-adjusted mortality declined substantially among men during the study period, mortality remained stable or slightly increased among women, resulting in a progressive narrowing of the sex gap in LC mortality. Formal temporal analysis for 2002–2024 showed that age-adjusted lung cancer mortality declined overall (APC −1.00%; 95% CI −1.30 to −0.71) and among men (APC −1.87%; 95% CI −2.17 to −1.55). Among women, there was no evidence of a significant overall temporal trend (APC 0.31%; 95% CI −0.04 to 0.66; *p* = 0.076); the dashed lines represent the fitted log-linear temporal trends used to estimate the APCs.

The total number of deaths during the study period was 2,772,043, of which 75,701 (2.7%) were due to lung cancer. The crude mortality rate increased from 12.0 to 17.8 per 100,000 people between 1997 and 2024; in contrast, the age- and sex-adjusted mortality rate decreased from 22.2 per 100,000 inhabitants in 1997 to 17.8 per 100,000 in 2024. Although rates remained relatively stable during the 2000s, a sustained decline became evident from approximately 2013 onward, reaching the lowest levels observed during the study period. Lung cancer mortality showed opposite trends by sex. Among men, the age-standardized rate decreased from 35.1 per 100,000 in 1997 to 22.6 per 100,000 in 2024, representing an approximate 35.5% reduction over the period. In contrast, among women, the age-standardized mortality rate showed a slight overall increase, rising from 12.9 to 14.1 per 100,000 equivalent to an increase of nearly 9%. Throughout the entire period, age-adjusted mortality rates remained consistently higher in men than in women, although the gap between the two sexes narrowed significantly (Figure 1, Table S2).

Mortality projections showed contrasting patterns by sex. Compared with 2021–2025, the projected number of deaths in 2031–2035 decreased from 9830 to 9670 among men, with the crude mortality rate declining from 19.97 to 18.68 per 100,000. Among women, projected deaths increased from 7723 to 9079, accompanied by an increase in the crude mortality rate from 15.28 to 17.07 per 100,000. Overall, approximately 18,501 lung cancer deaths were projected for 2031–2035, corresponding to a crude rate of 17.63 per 100,000.

### 3.2. Characterization of Lung Cancer Hospitalizations

**Figure 2:**
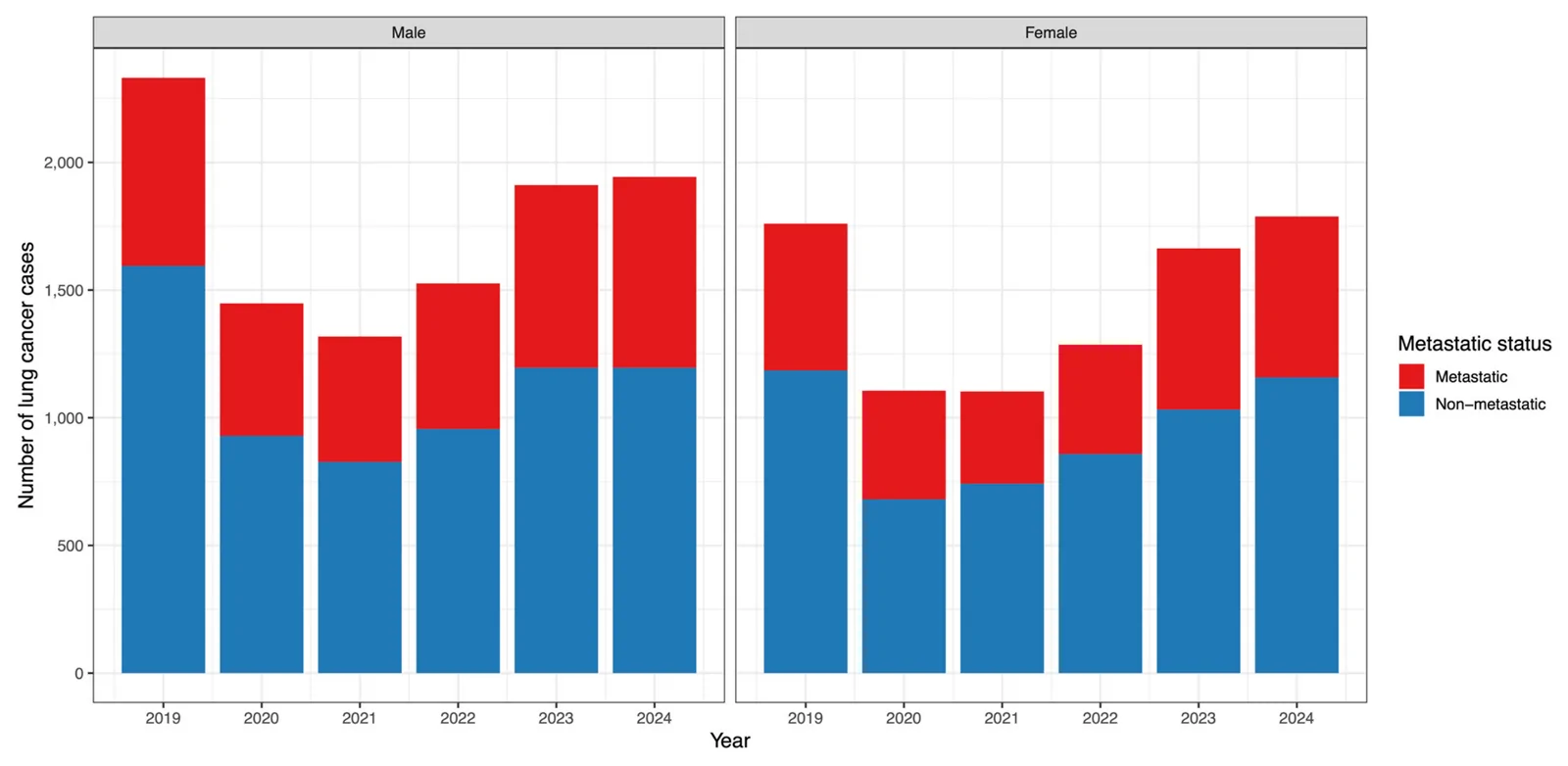
Annual number of hospitalizations related to lung cancer in Chile, 2019–2024. Annual number of hospitalizations among patients with lung cancer recorded in the national hospital discharge database (GRD-FONASA) between 2019 and 2024. A decrease in hospitalizations is observed during 2020–2021, followed by progressive recovery in subsequent years.

Between 2019 and 2024, 5,808,536 hospitalizations were identified in the GRD-FONASA hospital discharge database, of which 19,186 were related to lung cancer. The annual number of hospitalizations was 4092 in 2019, 2554 in 2020, 2421 in 2021, 2812 in 2022, 3574 in 2023, and 3733 in 2024 (Figure 2). The mean age of hospitalized patients was 67.3 years (SD 11.9). Men accounted for 54.6% of hospitalizations (n = 10,477). The mean age was similar between the two sexes (67.4 vs. 67.1 years). In total, 3335 hospitalizations resulted in death, corresponding to an in-hospital mortality rate of 17.4%.

Using the encrypted patient identifier, we identified 13,477 unique patients corresponding to the 19,186 hospitalizations recorded during the study period. Among these patients, 7377 were men (54.7%) and 6100 were women (45.3%). The mean number of hospitalizations per patient was 1.43, and 24.4% of patients experienced more than one hospitalization during the study period.

### 3.3. Reasons for Hospitalization and Differences by Outcome (Cancer/Metastasis, Pneumonia, Acute Complications)

**Figure 3:**
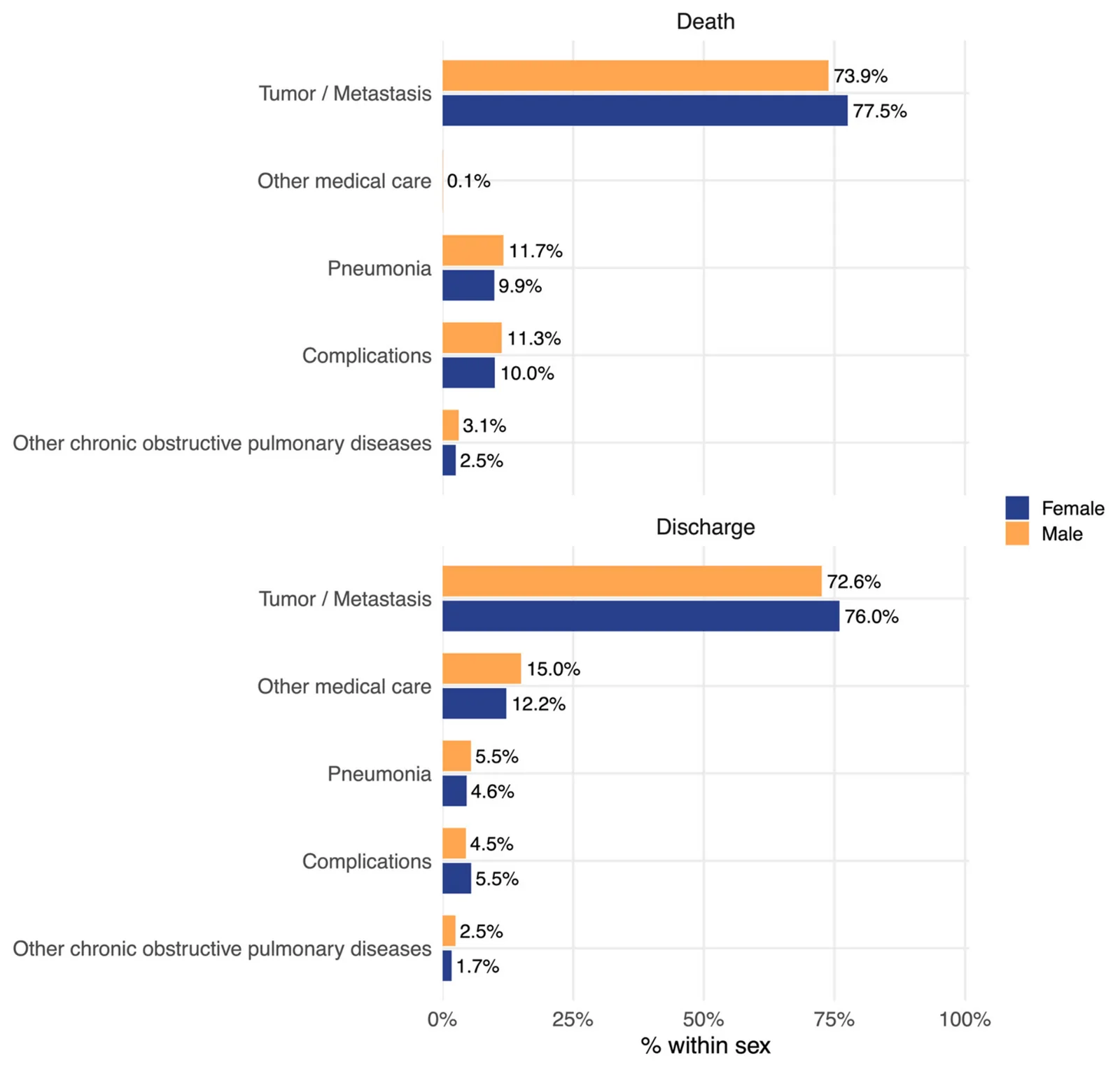
Distribution of main reasons for hospitalization among patients with lung cancer according to hospital outcome and sex. Proportion of hospitalizations according to the main diagnostic category among patients with lung cancer, stratified by hospital outcome (death vs. discharge) and sex.

Among the 14,778 hospitalizations included in the selected principal diagnosis analysis, lung cancer or metastasis was the most common principal diagnosis, accounting for 10,986/14,778 (74.3%) hospitalizations. This category accounted for 1122/1519 (73.9%) hospitalizations ending in death and 4798/6612 (72.6%) ending in live discharge among men, and 829/1069 (77.5%) and 4237/5578 (76.0%), respectively, among women. However, respiratory infections and acute pulmonary complications were relatively more common among hospitalizations with a fatal outcome. Among patients who died, pneumonia accounted for 177/1519 (11.7%) of cases in men and 106/1069 (9.9%) in women, while respiratory complications accounted for 172/1519 (11.3%) and 107/1069 (10.0%), respectively. These conditions were less common among hospital discharges without death (361/6612 [5.5%] and 257/5578 [4.6%] for pneumonia, and 295/6612 [4.5%] and 306/5578 [5.5%] for respiratory complications in men and women, respectively). The distribution of diagnoses according to hospital outcome was statistically significant in both men and women (chi-square test, *p* < 0.001 in both cases) (Figure 3).

The numbers of in-hospital deaths and total hospitalization episodes within each principal-diagnosis category, overall and by sex, are reported in Supplementary Table S3.

### 3.4. Overall In-Hospital Mortality

In-hospital mortality was higher among men than among women (18.7% vs. 15.8%). In the unadjusted logistic regression model, male sex was associated with higher odds of in-hospital death (OR 1.22; 95% CI 1.13–1.31; *p* < 0.001). This association persisted after adjustment for age (adjusted OR 1.22; 95% CI 1.13–1.31; *p* < 0.001). Age was also independently associated with in-hospital mortality, with each additional year of age associated with higher odds of death (OR 1.02; 95% CI 1.01–1.02; *p* < 0.001). In-hospital mortality varied over the study period, increasing from 15.0% in 2019 to a peak of 19.3% in 2020, remaining elevated in 2021 (18.5%) and 2022 (18.4%). Mortality subsequently decreased to 17.0% in 2023 and slightly increased to 17.6% in 2024.

#### Hospitalization Sequence and In-Hospital Mortality

Of the hospitalizations analyzed, 13,432 corresponded to first hospitalizations and 5751 to readmissions. In-hospital mortality was higher during first hospitalizations compared with readmissions (19.0% vs. 13.5%; χ^2^ test, *p* < 0.001). In multivariable logistic regression analysis adjusting for age and sex, lower odds of in-hospital death were observed among readmissions compared with first hospitalizations (OR 0.70; 95% CI 0.64–0.76). This comparison should be interpreted cautiously because readmission is conditional on surviving a previous hospitalization and may therefore be subject to survival and selection bias.

### 3.5. Multivariable Analysis of In-Hospital Mortality

**Figure 4:**
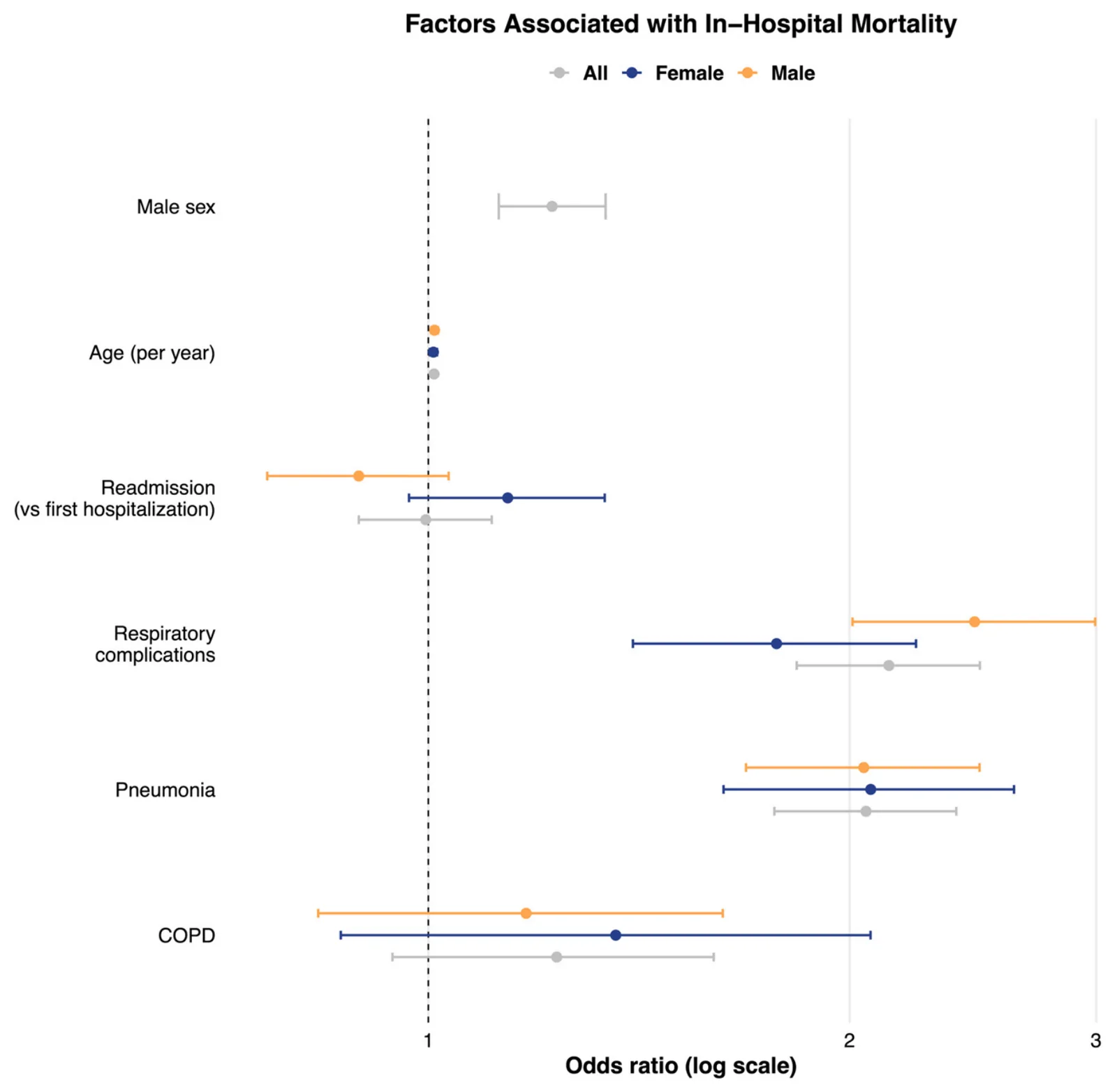
Factors associated with in-hospital mortality by sex. Forest plot of odds ratios (ORs) and 95% confidence intervals (CIs) from multivariable logistic regression models (overall and sex-stratified). The dashed line indicates OR = 1. “Other medical care” was excluded due to unstable estimates from sparse events (see Supplementary Tables S5 and S6).

To further evaluate factors associated with in-hospital mortality, a multivariable logistic regression model was constructed by additionally incorporating hospitalization characteristics, including hospitalization sequence and the main reason for hospitalization. After adjustment for these variables, the cause of hospitalization remained associated with in-hospital mortality. Compared with hospitalizations attributed to lung cancer or metastasis, those related to pneumonia were associated with more than a twofold higher odds of death (OR 2.05; 95% CI 1.76–2.38; *p* < 0.001). Likewise, hospitalizations due to acute respiratory complications were also associated with a higher odds of in-hospital death (OR 2.13; 95% CI 1.83–2.48; *p* < 0.001). Male sex remained independently associated with higher mortality (OR 1.23; 95% CI 1.12–1.34; *p* < 0.001), and each additional year of age was associated with a modest increase in the odds of death (OR 1.01; 95% CI 1.01–1.01; *p* < 0.001). In contrast, hospitalization sequence (readmission vs. first admission) was not associated with in-hospital mortality (OR 1.00; 95% CI 0.89–1.11; *p* = 0.939) (Figure 4).

Hospitalizations decreased by 37.6% in 2020 and 40.8% in 2021 compared with 2019. In a sensitivity analysis excluding 2020–2021, the associations with in-hospital mortality persisted for pneumonia (OR 1.81; 95% CI 1.52–2.16) and acute respiratory complications (OR 1.97; 95% CI 1.65–2.36), although the point estimates were lower than those observed in the full 2019–2024 analysis. In an analysis separating acute respiratory complications, respiratory failure (J96) was associated with higher odds of in-hospital death compared with the tumor/metastasis reference category (adjusted OR 7.438; 95% CI 5.785–9.563). In contrast, the estimates for pleural effusion (J90; adjusted OR 0.709; 95% CI 0.501–1.004) and pulmonary embolism (I26; adjusted OR 1.231; 95% CI 0.930–1.630) had confidence intervals that included the null value (Figure S2).

### 3.6. Sex-Stratified Analysis (Effect Modification)

In sex-stratified analyses, respiratory complications were associated with higher odds of in-hospital mortality in both women (OR 1.77; 95% CI 1.40–2.23) and men (OR 2.46; 95% CI 2.01–3.00). The point estimate was higher in men, but comparison of the stratified estimates alone does not establish effect modification by sex (Tables S4–S6).

## 4. Discussion

This population-based study is one of the first of its kind in Latin America to examine trends in lung cancer mortality in Chile over the past few decades and hospitalization patterns for this disease. Overall, the results show that, although age-adjusted lung cancer mortality has declined in Chile over the past few decades, this decline has been driven primarily by the decrease observed among men [13]. Similarly, the trend observed among women shows stable or slightly increasing rates over the study period, with data similar to those reported in the international literature [3]. The differences observed by sex may reflect multiple factors, such as the historical delay in the onset of smoking among women and in the implementation of tobacco control policies targeting this population [4], exposure to environmental carcinogens, limited access to screening and early diagnosis programs, and barriers to accessing high-quality cancer treatments [14,15,16]. Recent evidence further suggests that sex-specific lung cancer patterns may reflect a complex interplay of smoking patterns, environmental exposures, and biological factors [17,18]. These temporal differences in exposure patterns have led to a gradual shift in the burden of lung cancer mortality between men and women [5] and differences have also been observed in tumor characteristics among Chilean, Brazilian, and Peruvian women who have never smoked [17]. The projections further suggest that sex-specific differences may persist over the next decade, with declining mortality projected among men but increasing mortality among women. Sex-specific differences in future lung cancer burden have also been reported in international projections using the NORDPRED framework, which projected lung cancer incidence through 2035 across 40 countries [19]. These estimates should be interpreted as model-based projections rather than forecasts of observed future mortality. To limit the influence of long-term extrapolation, the projected trend was left unattenuated for 2026–2030 and reduced by 25% for 2031–2035. Nevertheless, these projections remain dependent on the continuation of historical patterns and may be affected by future changes in population structure, risk-factor exposure, screening, and treatment. The contrasting crude and age-standardized mortality trends may partly reflect population aging. Age-standardized rates therefore provide a more appropriate measure of temporal changes in lung cancer mortality.

At the hospital level, the episode-level in-hospital mortality observed in the GRD-FONASA data (17.4%) reflects the high clinical severity of patients hospitalized with lung cancer and is consistent with the literature, which describes a high burden of respiratory complications in this population [20]. In our analysis, male sex, older age, and the presence of respiratory infections or acute pulmonary complications were independently associated with a higher odds of in-hospital death. These associations should be interpreted cautiously, as adjustment for important clinical indicators of disease severity and prognosis was not possible. These findings are consistent with previous evidence, which shows that these patients are more susceptible to respiratory infections and serious pulmonary events [21] in a context where hospitalization typically occurs in the advanced stages of the disease, which increases the risk of clinical complications and mortality [6,22]. However, the administrative data do not allow differentiation between admissions for active cancer treatment, disease progression, or end-of-life/palliative care, which may represent distinct clinical contexts and mortality profiles.

The marked reduction in recorded hospitalizations during 2020–2021 coincided with the COVID-19 pandemic and may reflect pandemic-related changes in healthcare utilization. In a sensitivity analysis excluding these years, the associations between in-hospital mortality and both pneumonia and acute respiratory complications persisted, although their magnitude was attenuated. These findings suggest that the main associations were not restricted to the period with the largest reduction in recorded hospitalizations.

A particularly significant finding of this study is the association between respiratory infections and in-hospital mortality in patients with lung cancer. Hospitalizations for pneumonia and other acute pulmonary complications were associated with approximately twice the odds of in-hospital death. Although COPD was not independently associated with in-hospital mortality in our analysis, its coexistence with lung cancer remains clinically relevant given the substantial overlap between these conditions and their shared environmental and inflammatory pathways [23]. This finding is consistent with the literature, which identifies respiratory infections as a common cause of clinical decompensation in this population [8,10]. In particular, pneumonia and other pulmonary complications have been associated with severe clinical deterioration and adverse outcome [24]. However, given the observational nature of our study, these associations should not be interpreted as causal. Furthermore, analyses stratified by sex reveal differences in the magnitude of the associations between clinical factors and in-hospital mortality. In particular, the effect of complications was more pronounced in men compared with women, which could reflect differences in clinical severity, the burden of comorbidities, or the timing of care. Although this study does not allow for an in-depth exploration of these mechanisms, the findings highlight the potential relevance of sex when examining clinical outcomes in hospitalized patients with lung cancer.

Lung cancer or metastasis was the principal diagnosis in 10,986 of the 14,778 hospitalizations included in the selected diagnostic analysis (74.3%). Although previous studies have described frequent hospital admissions among patients with advanced lung cancer near the end of life, our diagnostic data alone do not establish disease stage or proximity to death. Cross-national studies have reported that between 40% and 60% of lung cancer patients die in acute care hospitals, with considerable variation depending on the availability of community-based and specialist palliative care services [25,26]. In Chile, despite recent legislative advances toward universal palliative care coverage and a relatively developed palliative care infrastructure by regional standards, formal hospice services remain limited and unevenly distributed, with approximately 13.4 specialist palliative care services per million inhabitants as of 2018 [27,28]. This context may partly explain the concentration of end-of-life care events within the acute hospital setting observed in our data. Future research should integrate clinical records or discharge intent codes, where available, to differentiate treatment-oriented from comfort-oriented admissions, a distinction that is critical for evaluating the appropriateness of hospital use and for planning palliative care resources in Chile.

From a public health perspective, these findings have significant implications for the organization and management of lung cancer in Chile. The high mortality rate and the proportion of hospitalizations associated with advanced disease underscore the need to strengthen strategies for prevention, early diagnosis, and timely treatment. In this context, it is essential to maintain and expand tobacco control policies, as well as to improve access to diagnosis and specialized care. Furthermore, the observed impact of respiratory infections on in-hospital mortality underscores the importance of their prevention and timely management in these patients. Given the factors mentioned above, the approach to lung cancer requires a comprehensive perspective that includes clinical diagnosis and treatment, public health strategies focused on tobacco prevention and control, and the reduction in exposure to environmental pollutants. Integrating these determinants is key to moving toward more effective and sustainable prevention strategies. Beyond the Chilean context, these findings may be relevant to other countries undergoing similar epidemiological transitions in lung cancer. The contrasting mortality trends observed between men and women, together with the substantial burden of in-hospital mortality and respiratory complications, highlight challenges that may also be relevant in settings with changing patterns of tobacco exposure and heterogeneous access to cancer care. Moreover, this study illustrates the value of routinely collected mortality and hospital administrative data for monitoring lung cancer burden and hospital outcomes in settings where detailed clinical cancer registries may be limited.

This study has several strengths that lend credibility to its findings. First, it draws on national administrative records of mortality and hospitalizations with population coverage, which made it possible to analyze lung cancer trends in Chile over an extended period and with a large sample size. Furthermore, the combined use of both data sources made it possible to address the phenomenon from two complementary perspectives: on the one hand, mortality trends at the population level, and on the other, the clinical patterns associated with hospitalizations in patients with this disease. However, the results must be interpreted with certain limitations in mind. Since these are administrative databases, the available clinical information is limited and does not include relevant variables such as tumor stage, histological subtype, smoking history, cancer treatment, performance status, metastatic burden, ICU admission, and relevant comorbidities. These unmeasured factors may influence both hospitalization characteristics and in-hospital mortality; therefore, residual confounding cannot be excluded and the observed associations should be interpreted with caution. In addition, the primary diagnosis recorded in administrative data may not necessarily represent the principal clinical cause of deterioration or death during hospitalization. Hospitalization sequence should be interpreted cautiously because readmissions are conditional on surviving a previous hospitalization, introducing potential survival and selection bias. Therefore, associations involving pneumonia and acute respiratory complications should be interpreted according to the recorded primary diagnosis and not as evidence that these conditions were the direct cause of death. Furthermore, in the analysis of hospitalizations, the unit of analysis was the hospitalization episode rather than the individual patient; consequently, some patients may have contributed more than one record during the analyzed period. Finally, although the FONASA GRD database covers approximately 80% of the Chilean population, the results do not reflect hospitalization patterns in the private healthcare system [29]. Given the observational design of the study, it is not possible to establish causal relationships between the variables analyzed and in-hospital mortality. Despite these limitations, the temporal scope of the analysis and the national coverage of the registries used provide a robust picture of the burden of lung cancer in Chile and of the clinical factors associated with mortality among hospitalized patients.

This study has an additional limitation: the administrative databases used do not include information on environmental exposures, such as air pollution or biomass smoke, which have been associated with lung cancer risk in other studies [30,31,32]. The extent to which these exposures may influence the outcomes observed in this analysis could not be assessed. Furthermore, the administrative nature of the GRD-FONASA database does not allow for determining the clinical intent of each hospitalization, specifically, whether it was undertaken with curative or active treatment goals, or as part of end-of-life care. This distinction is relevant, as hospitalizations for symptom management in terminal patients may have different clinical trajectories and mortality profiles than admissions for active oncological therapy. Severe acute complications, particularly respiratory failure, may reflect advanced clinical deterioration or terminal events. Their temporal relationship with mortality cannot be established; therefore, reverse causation cannot be excluded. The absence of intent-of-care coding in Chilean administrative health records represents a structural limitation for research on end-of-life patterns in this population.

## 5. Conclusions

This study provides relevant population-based evidence on lung cancer mortality trends in Chile and on in-hospital mortality among hospitalization episodes recorded in the GRD-FONASA database, highlighting sex differences in mortality and the association of respiratory complications with in-hospital mortality. These findings characterize relevant epidemiological and hospital patterns and should be interpreted within the limitations of administrative data.

## Data Availability

The data used in this study are publicly available from official Chilean government sources. Mortality data are available from the Department of Health Statistics and Information (DEIS), Ministry of Health, at https://deis.minsal.cl/. Hospital discharge data based on Diagnosis-Related Groups (DRG) are available from the Chilean National Health Fund (FONASA) Open Data Portal at https://datosabiertos.fonasa.cl/.

## Supplementary Materials

The following supporting information can be downloaded at: https://www.mdpi.com/article/10.3390/healthcare14213688/s1, Figure S1. Sex-specific mortality rates from lung cancer in Chile, 1997–2024. Crude and age-adjusted mortality rates for lung cancer in Chile between 1997 and 2024 stratified by sex. The figure illustrates long-term trends and highlights the diverging patterns between men and women; Figure S2. Sensitivity analysis of individual respiratory conditions and in-hospital mortality among hospitalizations of patients with lung cancer. Points indicate adjusted odds ratios and horizontal bars represent 95% confidence intervals for respiratory failure (J96), pleural effusion (J90), and pulmonary embolism (I26), entered as separate principal-diagnosis categories in a single logistic regression model. The reference category was tumor/metastasis (C34, C78, and C79). The model was adjusted for sex, age, and calendar year, with patient-clustered robust standard errors, and included 19,182 hospitalizations during 2019–2024. Table S1. ICD-10 codes and diagnostic categories used in the hospitalization analysis. Official ICD-10 category titles and their corresponding study diagnostic groups are presented. Hospitalizations were classified according to the principal diagnosis at the three-character ICD-10 category level. The tumor/metastasis group (C34, C78, and C79) served as the reference category in the logistic regression models. ICD-10, International Statistical Classification of Diseases, 10th Revision; COPD, chronic obstructive pulmonary disease; Table S2. Annual crude and age-standardized lung cancer mortality rates by sex in Chile, 1997–2024; Table S3. In-hospital deaths by principal-diagnosis category and sex among GRD-FONASA hospitalization episodes. Values are presented as deaths/total hospitalizations within each diagnostic category. The table includes the 14,778 hospitalization episodes in the selected principal-diagnosis analysis; counts refer to episodes rather than unique patients; Table S4. Logistic regression model evaluating the association between sex and in-hospital mortality among patients hospitalized with lung cancer. Results from logistic regression models evaluating the association between sex and in-hospital mortality. Odds ratios (OR) and 95% confidence intervals (95% CI) are reported for crude and age-adjusted models; Table S5. Factors associated with in-hospital mortality among women hospitalized with lung cancer. Multivariable logistic regression included age, readmission status, and principal-diagnosis category. Odds ratios (OR), 95% confidence intervals (CI), and *p* values are reported. First hospitalization and lung cancer or metastasis were the respective reference categories; Table S6. Factors associated with in-hospital mortality among men hospitalized with lung cancer. Multivariable logistic regression included age, readmission status, and principal-diagnosis category. Odds ratios (OR), 95% confidence intervals (CI), and *p* values are reported. First hospitalization and lung cancer or metastasis were the respective reference categories.
