# Supplementary material for: Lung Cancer Mortality and In-Hospital Outcomes in Chile: A Population-Based Cross-Sectional Study
Source: Healthcare (Basel). 2026 Oct 31;14(21):3688. doi: 10.3390/healthcare14213688 (PMC13762925; doi:10.3390/healthcare14213688)
Supplement: Supplementary file 1 [file healthcare-14-03688-s001.zip › healthcare-4534303-supplementary.pdf]

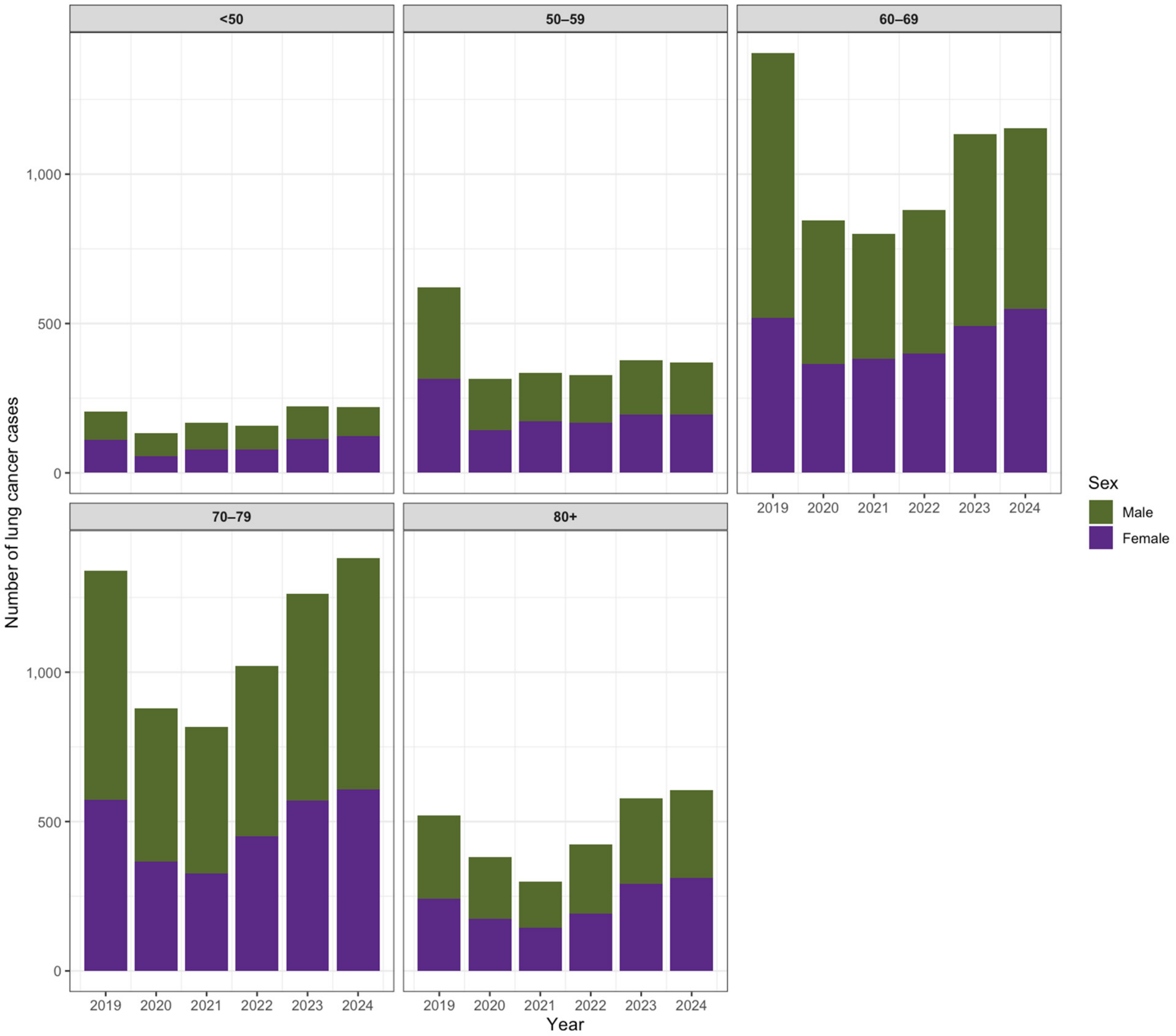

Figure S1. Sex-specific mortality rates from lung cancer in Chile, 1997-2024. Crude and age-adjusted mortality rates for lung cancer in Chile between 1997 and 2024 stratified by sex. The figure illustrates long-term trends and highlights the diverging patterns between men and women.

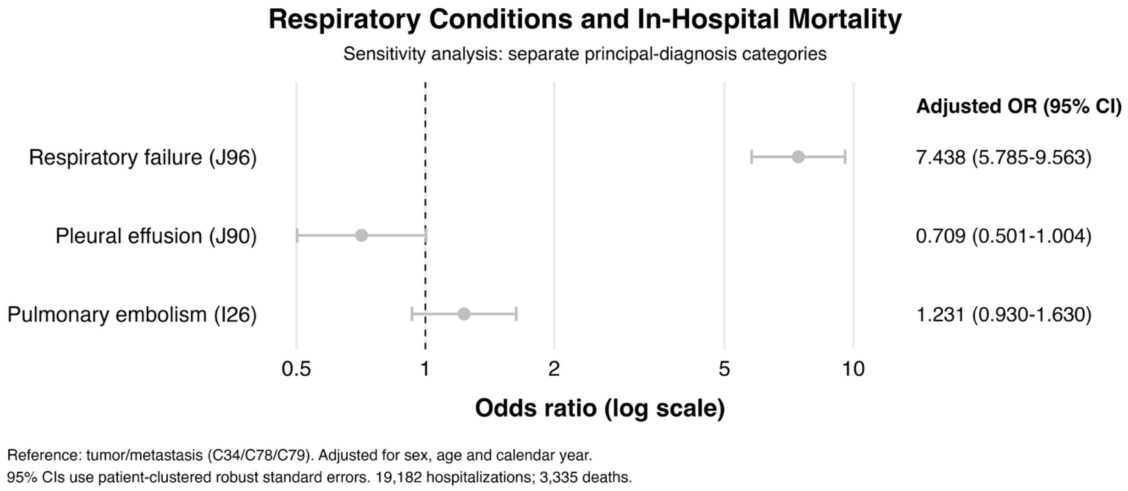

Figure S2. Sensitivity analysis of individual respiratory conditions and in-hospital mortality among hospitalizations of patients with lung cancer. Points indicate adjusted odds ratios and horizontal bars represent 95% confidence intervals for respiratory failure (J96), pleural effusion (J90), and pulmonary embolism (I26), entered as separate principal-diagnosis categories in a single logistic regression model. The reference category was tumor/metastasis (C34, C78, and C79). The model was adjusted for sex, age, and calendar year, with patient-clustered robust standard errors, and included 19,182 hospitalizations during 2019–2024. Readmission was not included.

Table S1. ICD-10 codes and diagnostic categories used in the hospitalization analysis. Official ICD-10 category titles and their corresponding study diagnostic groups are presented. Hospitalizations were classified according to the principal diagnosis at the three-character ICD-10 category level. The tumor/metastasis group (C34, C78, and C79) served as the reference category in the logistic regression models. ICD-10, International Statistical Classification of Diseases, 10th Revision; COPD, chronic obstructive pulmonary disease.

**Study diagnostic group**	**ICD-10 code**	**ICD-10 category title**
Tumor / Metastasis	C34	Malignant neoplasm of bronchus and lung
Tumor / Metastasis	C78	Secondary malignant neoplasm of respiratory and digestive organs
Tumor / Metastasis	C79	Secondary malignant neoplasm of other and unspecified sites

Pneumonia	J15	Bacterial pneumonia, not elsewhere classified
Pneumonia	J18	Pneumonia, organism unspecified
Respiratory complications	J96	Respiratory failure, not elsewhere classified
Respiratory complications	J90	Pleural effusion, not elsewhere classified
Respiratory complications	I26	Pulmonary embolism
COPD	J44	Other chronic obstructive pulmonary disease
Other medical care	Z51	Other medical care

Table S2. Annual crude and age-standardized lung cancer mortality rates by sex in Chile, 1997–2024.

Year	Men: crude rate	Men: age-standardized rate (95% CI)	Women: crude rate	Women: age-standardized rate (95% CI)	Total: crude rate	Total: age-standardized rate (95% CI)
1997	16.3	35.1 (33.0–37.3)	7.8	12.9 (11.9–14.0)	12.0	22.2 (21.1–23.3)
1998	16.8	35.2 (33.2–37.3)	7.3	11.6 (10.7–12.7)	12.0	21.5 (20.5–22.6)
1999	16.7	33.9 (31.9–35.9)	7.8	12.4 (11.4–13.4)	12.1	21.5 (20.5–22.5)
2000	16.8	34.0 (32.1–36.0)	7.9	12.3 (11.3–13.3)	12.3	21.3 (20.3–22.3)
2001	17.4	33.9 (32.1–35.9)	8.5	12.9 (11.9–13.9)	12.9	21.8 (20.9–22.8)
2002	17.4	33.4 (31.6–35.4)	8.8	13.0 (12.0–14.0)	13.0	21.7 (20.7–22.7)
2003	16.7	31.5 (29.8–33.4)	8.7	12.6 (11.7–13.6)	12.7	20.5 (19.7–21.5)
2004	18.5	33.7 (31.9–35.5)	9.4	13.2 (12.2–14.2)	13.8	21.9 (20.9–22.8)
2005	18.5	33.0 (31.3–34.8)	10.0	13.7 (12.7–14.6)	14.2	21.8 (21.0–22.8)
2006	19.1	33.9 (32.2–35.7)	10.5	14.0 (13.1–15.0)	14.7	22.3 (21.4–23.3)
2007	19.5	33.1 (31.5–34.9)	11.0	14.5 (13.6–15.5)	15.2	22.4 (21.5–23.3)
2008	18.4	30.7 (29.1–32.3)	11.0	14.1 (13.2–15.0)	14.6	21.2 (20.3–22.0)
2009	19.0	31.1 (29.6–32.8)	11.0	13.8 (12.9–14.7)	15.0	21.1 (20.3–22.0)
2010	20.1	32.1 (30.6–33.7)	11.3	13.8 (12.9–14.7)	15.6	21.6 (20.8–22.4)
2011	20.2	31.6 (30.1–33.1)	12.1	14.5 (13.6–15.4)	16.1	21.7 (20.9–22.6)
2012	19.6	29.8 (28.4–31.3)	13.2	15.5 (14.6–16.4)	16.3	21.6 (20.8–22.4)
2013	20.8	30.9 (29.5–32.4)	13.7	15.6 (14.7–16.5)	17.2	22.1 (21.4–22.9)
2014	20.8	30.3 (29.0–31.8)	13.6	15.2 (14.3–16.1)	17.1	21.7 (20.9–22.5)
2015	20.9	29.6 (28.2–31.0)	13.8	15.2 (14.3–16.0)	17.3	21.3 (20.6–22.1)
2016	20.9	29.1 (27.8–30.5)	14.0	15.0 (14.2–15.8)	17.4	21.0 (20.3–21.7)
2017	20.9	28.4 (27.2–29.8)	14.7	15.5 (14.7–16.3)	17.7	20.9 (20.2–21.7)
2018	21.2	28.2 (27.0–29.5)	14.2	14.6 (13.9–15.4)	17.6	20.4 (19.7–21.1)

2019	20.7	27.1 (25.9–28.3)	14.6	14.9 (14.1–15.6)	17.6	20.1 (19.4–20.7)
2020	20.6	26.5 (25.3–27.7)	14.3	14.4 (13.6–15.1)	17.4	19.5 (18.9–20.2)
2021	19.4	24.3 (23.2–25.4)	14.1	13.8 (13.0–14.5)	16.7	18.3 (17.7–18.9)
2022	20.0	24.3 (23.2–25.4)	15.2	14.4 (13.7–15.2)	17.5	18.6 (18.0–19.2)
2023	19.6	23.0 (22.0–24.1)	15.6	14.4 (13.7–15.2)	17.6	18.1 (17.5–18.7)
2024	19.9	22.6 (21.7–23.7)	15.7	14.1 (13.4–14.8)	17.8	17.8 (17.2–18.4)

Table S3. In-hospital deaths by principal-diagnosis category and sex among GRD-FONASA hospitalization episodes. Values are presented as deaths/total hospitalizations within each diagnostic category. The table includes the 14,778 hospitalization episodes in the selected principal-diagnosis analysis; counts refer to episodes rather than unique patients.

Diagnostic category	Women, deaths/hospitalizations	Men, deaths/hospitalizations	Total deaths/hospitalizations
Lung cancer or metastasis	829/5,066	1,122/5,920	1,951/10,986
Pneumonia	106/363	177/538	283/901
Respiratory complications	107/413	172/467	279/880
COPD	27/124	47/211	74/335
Other medical care	0/681	1/995	1/1,676
Total	1,069/6,647	1,519/8,131	2,588/14,778

Table S4. Logistic regression model evaluating the association between sex and in-hospital mortality among patients hospitalized with lung cancer. Results from logistic regression models evaluating the association between sex and in-hospital mortality. Odds ratios (OR) and 95% confidence intervals (95% CI) are reported for crude and age-adjusted models.

Variable	OR	95% CI	P value
Male sex (Ref: Female)	1.22	1.13 - 1.31	<0.001
Age	1.015	1.012 - 1.019	<0.001

Table S5. Factors associated with in-hospital mortality among women hospitalized with lung cancer. Multivariable logistic regression included age, readmission status, and principal-diagnosis category. Odds ratios (OR), 95% confidence intervals (CI), and P values are reported. First hospitalization and lung cancer or metastasis were the respective reference categories.

Variable	OR	95% CI	P value
Age (per year)	1.01	1.00-1.014	0.006
Readmission (vs first hospitalization)	1.14	0.97-1.34	0.112
Respiratory complications	1.77	1.4-2.23	<0.001
Pneumonia	2.07	1.63-2.62	<0.001
COPD	1.36	0.87-2.07	0.164

Table S6. Factors associated with in-hospital mortality among men hospitalized with lung cancer. Multivariable logistic regression included age, readmission status, and principal-diagnosis category. Odds ratios (OR), 95% confidence intervals (CI), and P values are reported. First hospitalization and lung cancer or metastasis were the respective reference categories.

Variable	OR	95% CI	P value
Age (per year)	1.01	1.005-1.02	<0.001
Readmission (vs first hospitalization)	0.89	0.77-1.03	0.133
Respiratory complications	2.48	2.01-3	<0.001
Pneumonia	2.05	1.69-2.48	<0.001
COPD	1.18	0.83-1.62	0.342
Other medical care	0,00480	0,00027–0,02125	<0.001
